# On-Orbit Calibration of Installation Matrix between Remote Sensing Camera and Star Camera Based on Vector Angle Invariance

**DOI:** 10.3390/s20195667

**Published:** 2020-10-04

**Authors:** Yujie Tang, Zhenzhong Wei, Xinguo Wei, Jian Li, Gangyi Wang

**Affiliations:** School of Instrumentation and Opto-electronic Engineering, Beihang University, Beijing 100191, China; qianqiankun_tang@buaa.edu.cn (Y.T.); zhenzhongwei@buaa.edu.cn (Z.W.); wxg@buaa.edu.cn (X.W.); wanggangyi@buaa.edu.cn (G.W.)

**Keywords:** geo-positioning accuracy, vector angle invariance, star camera

## Abstract

To achieve photogrammetry without ground control points (GCPs), the precise measurement of the exterior orientation elements for the remote sensing camera is particularly important. Currently, the satellites are equipped with a GPS receiver, so that the accuracy of the line elements of the exterior orientation elements could reach centimeter-level. Furthermore, the high-precision angle elements of the exterior orientation elements could be obtained through a star camera which provides the direction reference in the inertial coordinate system and star images. Due to the stress release during the launch and the changes of the thermal environment, the installation matrix is variable and needs to be recalibrated. Hence, we estimate the cosine angle vector invariance of a remote sensing camera and star camera which are independent of attitude, and then we deal with long-term on-orbit data by using batch processing to realize the accurate calibration of the installation matrix. This method not only removes the coupling of attitude and installation matrix, but also reduces the conversion error of multiple coordinate systems. Finally, the geo-positioning accuracy in planimetry is remarkably higher than the conventional method in the simulation results.

## 1. Introduction

A high-resolution satellite image (HRSI) is important for high-precision geospatial information. It is widely used in many fields such as 3D shoreline extraction and coastal mapping, Digital Terrain Model (DTM) and Digital Surface Model (DSM) generation and national topographic mapping. Many of the above remote sensing applications require an HRSI with high accuracy [1]. The HRSI reconstruction method is mainly divided into four steps: feature extraction, feature matching, exterior orientation element estimation and final resampling [2,3,4]. For the high-precision exterior orientation element estimation, it is the key to realizing high-precision geo-positioning.

In geometric photogrammetry, when the image coordinate of the remote sensing image is determined, the geo-positioning depends on the accuracy of the provided external orientation element. The line element is obtained by the GPS receiver, the angle element can be obtained by the star camera or star sensor in the earth center inertial coordinate system [5]. Then, we get the angle element of the remote sensing camera by calculating the installation relationship between the remote sensing camera and star camera or star sensor (hereinafter called the installation matrix instead). Before the satellite launches, satellite designers measure the installation matrix on the ground. Due to the stress release during the launch and the changes of the thermal environment, the changed installation matrix will result in the failure of geo-positioning. So, it requires on-orbit calibration.

The early published works in this area usually seem to use the remote sensing camera to obtain its exterior orientation elements through the GCPs. The attitude determination equipment outputs the satellite’s attitude. Then, the installation matrix could be solved [6]. After the launch of the SPOT-5 satellite in 2002, the French Space Center established the angle error model of the optical axis between the star sensor and remote sensing camera. They used the global distribution of test sites to calculate the boresight direction of the remote sensing camera, then they calculated the error of the installation matrix with the attitude and orbit control subsystem (AOCS). Furthermore, the error model of the internal orientation elements was proposed which was fitted by the fifth polynomial. Finally, the positioning accuracy of SPOT-5 single scene without GCP could reach 50 m [7]. This method establishes the basic on-orbit calibration process of the installation matrix, but it does not research the requirements of GCPs and the real orientation of each pixel [8]. Dial G and Grodecki J establish a joint calibration of the IKONOS satellite based on the Field Angle Map (FAM) and the interlock angle between optical axis of the remote sensing camera and star sensor. FAM could solve the real orientation of each pixel in CCDs. Then, they use the knife-edge targets in a set of independent images to calibrate interlock angle errors, and after that, they compute the mean interlock angle correction. Finally, the accuracy can reach 12 m (RMS) in planimetry and 10 m (RMS) in elevation without GCP [9,10]. This analysis establishes the angle model and puts forward requirements for the types of GCPs, which makes the identification and extraction of GCPs more accurate and visible. However, the method of obtaining the exterior orientation elements of the remote sensing camera directly through the GCPs will lead to a strong correlation of the azimuth parameters, which leads to errors in the solution of the installation matrix [11].

In order to reduce the strong coupling of azimuth parameters, the following published works report the methods which are based on Taylor expansion and an iterative solution of a remote sensing camera’s angle elements. They thought of the angle elements as unknown quantities, which are expanded in the Taylor series at the center of the scene and solved iteratively in the strict imaging model. Yuan X. X thought there was the constant difference which was the error of the remote sensing image point direction and the true direction. The image point direction is calculated from the image attitude, and the true direction is the direction which is from the satellite to the GCP. This constant difference is the deviation of the initial installation matrix. Through the line of sight consistency of a small number of GCPs in the geocentric rectangular coordinate system and image coordinate system, the deviation matrix can be calculated and then compensated. After they compensated the installation matrix of the QuickBird satellite, the positioning accuracy without GCPs can increase from 8.59 to 3.09 m [12]. Wang Q. L. used the similar method which established several collinear equations through the GCPs, and he calculated the installation matrix by the initial values of ground calibration. This work was efficient because he could obtain the stable exterior orientation elements through the iteration of the internal and external parameters calculation. Then, the installation matrix could be calculated easily. Each GCP can get an installation matrix. He acquired the optimal installation matrix by establishing the optimization objective equation. Finally, the RMS deviation reached 9.3 m [13]. For the two methods above, these methods can solve the attitude of the remote sensing camera by Taylor expansion to reduce the dependence on the satellite attitude determination system, but they cannot avoid the coupling problem between the random error of the attitude and the installation matrix [14]. At the same time, for the satellite attitude determination system and the remote sensing camera work that is unsynchronized, the fitting and interpolation calculation of external parameters also brings errors [15].

In this paper, an improved geo-positioning of satellite imagery is proposed to meet the requirements of high accuracy observation. There are three advantages for using this method. First, we introduce the concept of the star camera, which is an optical load of the remote sensing camera’s platform. The star camera combines two functions: one is autonomous navigation attitude measurement such as the star sensor, and the other is the external orientation elements acquirement of the remote sensing camera’s current image. In order to avoid fitting and interpolation errors caused by the satellite attitude determination system and the remote sensing camera, they work unsynchronized. The star camera and the remote sensing camera shoot simultaneously through the synchronization signal, the star camera provides the real-time attitude, and then it calculates the exterior orientation elements of the remote sensing camera through the installation matrix. In terms of camera models, we use the rigorous imaging model (the sensor model of multi-line array CCD can refer to Section 3). Second, unlike many coordinate system transformations in previous algorithms, this method only involves simple conversion between the World Geodetic System 1984 (WGS84), the earth-centered inertial reference frame (ECI, here is J2000), the star camera, and the remote sensing camera coordinate system. It does not need to consider the satellite motion in the orbital coordinate system. We only use the GPS position in real-time, image data of remote sensing camera and star camera in synchronization time to complete the high-precision geo-positioning. Third, unlike the existing algorithms which need to calculate the star sensor and remote sensing camera’s own attitude matrix, the input calibration data only consist of a star vector and ground calibration target vector (GCT vector). Because the number of star vectors captured by the star camera is large, it can reduce the dependence on the number of GCTs. For details, please see Section 2.

The remainder of this paper is organized as follows. A brief introduction of this method is given in Section 2. Section 3 describes the simulation process and the results of the algorithm, including building an imaging model, simulation database preparation, and the verification of simulation calculation in detail. Section 4 presents some conclusions.

## 2. Algorithm

In this part, we propose an algorithm, which is not only needed to use the complex sensor model design of a rigorous sensor model, but also can reduce the coordinate system transformations and demand of traditional on-orbit calibration for the number of GCTs. The core of this method is: when the remote sensing camera’s external orientation elements are determined, the vector angles between the GCT vector (the GCT is the projection of corresponding image points on the ground) and star vector are the rotation invariances. Then, the installation matrix is calculated by the method of the double-vector attitude determination and optimized in real-time by batch processing on orbit.

The method’s steps are as follows: the remote sensing image is acquired by a synchronous pulse, and the star point image is also acquired by the star camera at the same time. The corresponding relationship between the remote sensing image characteristic points and the GCTs are obtained through the feature extraction and recognition. When the GCTs are located, the GCT vectors are obtained from the satellite position (the direction of the vectors is from the satellite body pointing to the GCTs). Because the satellite’s external orientation elements (position and attitude) are known, the vector angles between the GCT vector and the star vector can be calculated precisely. The GCT vectors in the remote sensing image coordinate system and the star vectors in the star camera coordinate system also could be calculated. So, the installation matrix can be calibrated by these invariances.

**Figure 1:**
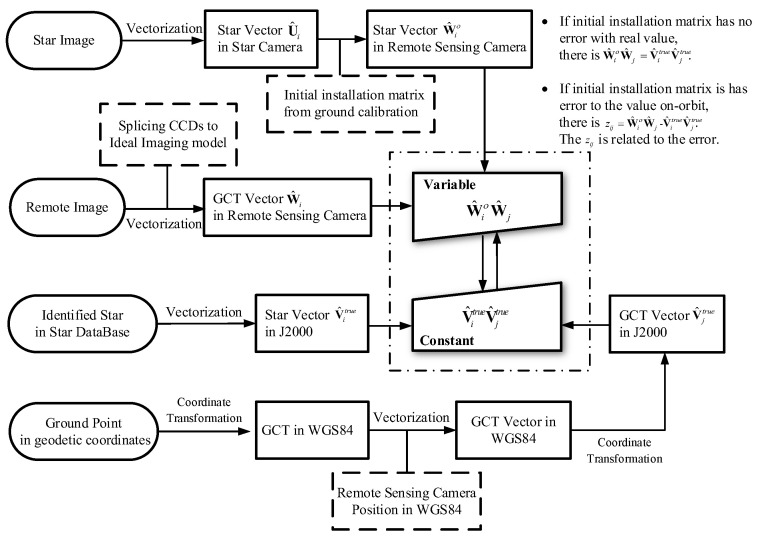
Method Flow Chart.

Figure 1 is a flow chart of the paper method. We can clearly see that there is no estimation of the motion state of the remote sensing camera in the method. When the position of the remote sensing camera is known, the coordinates of the ground point and the identified star point are all accurately known, so ${\hat{V}}_{i}^{true}{\hat{V}}_{j}^{true}$ is the constant depends on remote sensing camera position. If the installation matrix is known precisely, ${\hat{W}}_{i}^{ο}{\hat{W}}_{j}^{}$ is equal to ${\hat{V}}_{i}^{true}{\hat{V}}_{j}^{true}$ in the ideal imaging model. However, because of the existence of the initial installation matrix error, camera distortion residual, model error and others, the variable ${\hat{W}}_{i}^{ο}{\hat{W}}_{j}^{}$ is approximately equal to the constant ${\hat{V}}_{i}^{true}{\hat{V}}_{j}^{true}$. Through the method in this paper, we associate the error of the installation matrix with the optimization equation composed of ${\hat{W}}_{i}^{ο}{\hat{W}}_{j}^{}$ and ${\hat{V}}_{i}^{true}{\hat{V}}_{j}^{true}$, and the estimation error of input data. Finally, we optimize the error of the installation matrix by using the coordinates of stars and the corresponding GCTs.

### 2.1. Mathematical Model

To obtain the ground coordinate on earth from the remote sensing image coordinate, it is necessary to use a mathematical model to describe the relationship between the two sets of coordinates. Each GCT will provide a set of two collinear condition equations derived from image coordinates, satellite position and GCT coordinates in the conventional inertial system (in the field of aerospace, we usually use J2000 as the conventional inertial system, so the conventional inertial system is hereinafter referred to as J2000) [16,17].

(1) $$\left[\begin{array}{c} 0\\ y-y_{0}\\ -f \end{array}\right]=\lambda M\left[\begin{array}{c} X-X_{P}\\ Y-Y_{P}\\ Z-Z_{P} \end{array}\right]$$

Now, describe the components of this Equation (1). $y_{}$ is the image coordinate, $y_{0}$ is the principal point of the image (the principal point is the point where the optical axis intersects the image plane). M is an orthogonal rotation matrix that represents the transformation from J2000 to the image sensor coordinate system. The origin of the remote sensing camera coordinate system is located in the perspective center, the *Y*-axis is parallel to the detector array, the *X*-axis points to the pushbroom direction and the *Z*-axis is perpendicular to the plane of X and Y axis while pointing to the observation target. f is the focal length of star camera. λ is a scale factor. (X,Y,Z) is the ground point’s coordinate in the J2000. We usually get the ground points based on local geodetic data but calculate in J2000. There are three steps to coordinate system transformation. First, correct the height of geoid to separate geoid and ellipsoid. Then, the coordinates are transformed from a geodetic coordinate system to a geocentric coordinate system in local data. Third, the geocentric coordinates system can be converted from local to WGS84. Finally, WGS84 coordinates are transformed into the J2000 coordinate system. $\left(X_{P},Y_{P},Z_{P}\right)$ is the perspective center coordinate of remote sensing camera in the J2000.

The M matrix changes with time t. It can be expressed as:

(2) $$M=R_{CS}\cdot R_{SI}$$

where $R_{CS}$ is the installation matrix between star camera and remote sensing camera. $R_{SI}$ is the rotation matrix from the J2000 to the star camera coordinate system, also named the attitude matrix. $R_{SI}$ is given by the star camera. $R_{CS}$ is obtained by ground calibration. For the stress release during the launch and the change in the working environment, there is a deviation from the initial value, so $R_{CS}$ needs to be calibrated on orbit.

The observation star vector of the star camera satisfies the condition as:

(3) $$u=R_{SI}^{}v$$

u is the representation of the star vector in the star camera coordinate system, and v is the representation in the J2000.

Where

$$u=\frac{1}{\sqrt{{\left(\bar{x}\right)}^{2}+{\left(\bar{y}\right)}^{2}+f^{2}}}\left[\begin{array}{c} -\bar{x}\\ -\bar{y}\\ f \end{array}\right],v=\left[\begin{array}{c} \cos\delta \cos\alpha \\ \cos\delta \sin\alpha \\ \sin\delta \end{array}\right]$$

the right ascension and declination of star in the star catalog are expressed as α and δ. The above equation is based on the ideal imaging model. In fact, there will be lens distortion in star cameras, and we get the Equation (4).

(4) $$\{\begin{array}{c} X^{\prime }=X+dx\\ Y^{\prime }=Y+dy \end{array}$$

(X,Y) are the image coordinates of target in ideal imaging model, and $\left(X^{\prime },Y^{\prime }\right)$ are the actual imaging coordinates. (dx,dy) is the distortion value.

(5) $$\{\begin{array}{c} dx=\bar{x}\left(q_{1}r^{2}+q_{2}r^{4}\right)+\left[p_{1}\left(r^{2}+2{\bar{x}}^{2}\right)+2p_{2}\bar{x}\bar{y}\right]\\ dy=\bar{y}\left(q_{1}r^{2}+q_{2}r^{4}\right)+\left[p_{2}\left(r^{2}+2{\bar{y}}^{2}\right)+2p_{1}\bar{x}\bar{y}\right] \end{array}$$

(6) $$\{\begin{array}{l} \bar{x}=X-X_{0}\\ \bar{y}=Y-Y_{0}\\ r^{2}={\bar{x}}^{2}+{\bar{y}}^{2} \end{array}$$

$\left(X_{0},Y_{0}\right)$ is the principal point of the star camera. (q_1_, q_2_) is the radial distortion, and (p_1_, p_2_) is the tangential distortion.

### 2.2. Equation Derivation of $R_{CS}$

The star vector in the coordinate system of the remote sensing camera can be expressed as w, then:

(7) $$w=R_{CS}^{}u=R_{CS}^{}R_{SI}^{}v$$

We use Equations (1)–(3) and (7) to calculate the vector angles between GCT vectors and star vectors in the same coordinate system.

To avoid the influence of the attitude, the angle cosine which is independent of the attitude is selected for estimation. We deduce the relationship between the measured parameters (including the original vectors and the angle cosine), and we explain the non-simultaneous observation properties between the attitude and the absolute installation matrix. Based on the statistical characteristics of the measured parameters, the maximum likelihood estimation of the relative attitude was derived. Then, the redundancy of the measured parameters is discussed, and the decomposition algorithm is used to obtain the maximum independent measurement subset.

The star vectors ${\hat{U}}_{i,k}$ in the star camera coordinate system which can be observed are expressed as:

(8) $${\hat{U}}_{i,k}={\hat{U}}_{i,k}^{\mathrm{true}}+\Delta {\hat{U}}_{i,k}$$

${\hat{U}}_{i,k}^{\mathrm{true}}$ is the true value of the star vector, $\Delta {\hat{U}}_{i,k}$ is the measurement noise, i is the vector number, $i=1,2,\cdots ,n_{k}$, k is the time series, k=1,2,⋯,N. 

Similarly, the observation vectors (here means GCT vectors or star vectors after coordinate system conversion) ${\hat{W}}_{i,k}$ in the remote sensing camera coordinate system can be expressed as:

(9) $${\hat{W}}_{i,k}={\hat{W}}_{i,k}^{\mathrm{true}}+\Delta {\hat{W}}_{i,k}$$

Here, the same observation vectors in remote sensing camera and star camera coordinate system are expressed as follows:

(10) $${\hat{U}}_{i,k}=R_{CS}^{T}{\hat{W}}_{i,k}=R_{CS}^{T}{\hat{W}}_{i,k}^{\mathrm{true}}+R_{CS}^{T}\Delta {\hat{W}}_{i,k}\equiv {\hat{U}}_{i,k}^{\mathrm{true}}+\Delta {\hat{U}}_{i,k}$$

In general, the installation matrix is not known for its true value, and the initial value $R_{CS}^{0}$ can only be obtained by ground calibration before launch. Therefore, the error matrix is defined like this:

(11) $$R_{CS}^{}=MR_{CS}^{0}$$

Define the error vector θ, which has the following relationship with the error matrix M [18]:

(12) $$M\equiv e^{〚\theta 〛}=I+\left(\frac{\sin\left|\theta \right|}{\left|\theta \right|}\right)〚\theta 〛+\left(\frac{1-\cos\left|\theta \right|}{{\left|\theta \right|}^{2}}\right){〚\theta 〛}^{2}$$

Among them, $I=\left[\begin{array}{ccc} 1 & 0 & 0\\ 0 & 1 & 0\\ 0 & 0 & 1 \end{array}\right]$, $e^{\left\{\cdot \right\}}$ is matrix series, $〚\theta 〛\equiv \left[\begin{array}{ccc} 0 & \theta_{3} & -\theta_{2}\\ -\theta_{3} & 0 & \theta_{1}\\ \theta_{2} & -\theta_{1} & 0 \end{array}\right]$, $\theta_{1},\theta_{2},\theta_{3}$ are structural error angles which are usually small.

Here, we can simplify this equation:

(13) $$M=I+〚\theta 〛+O({\left|\theta \right|}^{2})\approx I+〚\theta 〛$$

So, we can the Equation (10) in first-order approximation:

(14) $${\hat{U}}_{i,k}=R_{CS}^{οT}M^{T}{\hat{W}}_{i,k}^{\mathrm{true}}+\Delta {\hat{U}}_{i,k}$$

Set ${\hat{V}}_{i}$ as the reference vector which is the expression of the observation vector of remote sensing camera in J2000.

(15) $${\hat{W}}_{i,k}^{\mathrm{true}}=R_{CI}{\hat{V}}_{i}^{\mathrm{true}}=R_{CI}{\hat{V}}_{i}-R_{CI}\Delta {\hat{V}}_{i}$$

$R_{CI}$ is the rotation matrix from J2000 to the remote sensing camera coordinate system, $\Delta {\hat{V}}_{i}$ is the reference vector error and, supposing it is gaussian, white noise with zero mean $E\{\Delta {\hat{V}}_{i}\Delta {\hat{V}}^{T}{}_{i}\}=R_{{\hat{V}}_{i}}$.

Because the error of the reference vector is too small, it is generally negligible. Therefore, the relationship between the star vectors and the reference vectors is expressed as:

(16) $${\hat{U}}_{i,k}=R_{CS}^{T}R_{CI}{\hat{V}}_{i}+\Delta {\hat{U}}_{i,k}=R_{CS}^{οT}M^{T}R_{CI}{\hat{V}}_{i}+\Delta {\hat{U}}_{i,k}$$

As can be seen from the above equation, the value of the star vectors ${\hat{U}}_{i,k}$ does not change for the following transformations:

(17) $$\begin{array}{l} R_{CS}^{}\to TR_{CS}^{}\left(MR_{CS}^{0}\to TMR_{CS}^{0}\right)\\ R_{CI}\to TR_{CI} \end{array}$$

T is an arbitrary rotation matrix. Therefore, it is impossible to get the installation matrix deviation from the attitude measurement of the star camera, so it also means estimating the sensor installation and the attitude cannot be achieved at the same time.

In this point, we introduce a new variable ${\hat{W}}_{i,k}^{ο}$ which means observation vectors in the uncalibrated remote sensing camera coordinate system.

(18) $${\hat{W}}_{i,k}^{ο}\equiv R_{CS}^{ο}{\hat{U}}_{i,k}=M_{}^{T}{\hat{W}}_{i,k}\approx M_{}^{T}R_{CI}{\hat{V}}_{i,k}+\Delta {\hat{W}}^{ο}{}_{i,k}$$

We expand the M matrix like this: M≈I+〚θ〛, so:

(19) $${\hat{W}}_{i,k}^{ο}\approx (I-〚\theta 〛){\hat{W}}_{i,k}^{\mathrm{true}}+\Delta {\hat{W}}^{ο}{}_{i,k}={\hat{W}}_{i,k}^{\mathrm{true}}+〚{\hat{W}}_{i,k}^{\mathrm{true}}〛\theta +\Delta {\hat{W}}^{ο}{}_{i,k}$$

It can be known from the equation that the ${\hat{W}}_{i,k}^{ο}$, s first-order approximation is still associated with the attitude through ${\hat{W}}_{i,k}^{\mathrm{true}}$.

(20) $${\hat{W}}_{i,k}^{ο}\cdot {\hat{W}}_{j,k}^{}={\hat{V}}_{i}^{true}\cdot {\hat{V}}_{j}^{true}+\left(-{\hat{W}}_{i,k}^{\mathrm{true}}\times {\hat{W}}_{j,k}^{true}\right)\cdot \theta +{\hat{W}}_{i,k}^{\mathrm{true}}\cdot \Delta {\hat{W}}_{j,k}^{}+\Delta {\hat{W}}_{i,k}^{ο}\cdot {\hat{W}}_{j,k}^{\mathrm{true}}+\Delta {\hat{W}}_{i,k}^{ο}\cdot \Delta {\hat{W}}_{j,k}^{}$$

$\Delta {\hat{W}}_{i,k}^{ο}\cdot \Delta {\hat{W}}_{j,k}^{}$ is the minterm. So, we make $z_{ij,k}$ and $\Delta z_{ij,k}$:

(21) $$\begin{array}{cc} z_{ij,k} & \equiv {\hat{W}}_{i,k}^{ο}\cdot {\hat{W}}_{j,k}^{}-{\hat{V}}_{i}^{true}\cdot {\hat{V}}_{j}^{true}\\ & =\left(-{\hat{W}}_{i,k}^{\mathrm{true}}\times {\hat{W}}_{j,k}^{true}\right)\cdot \theta +\Delta z_{ij,k}\approx \left(-{\hat{W}}_{i,k}^{ο}\times {\hat{W}}_{j,k}^{}\right)\cdot \theta +\Delta z_{ij,k} \end{array}$$

(22) $$\Delta z_{ij,k}={\hat{W}}_{i,k}^{\mathrm{true}}\cdot \Delta {\hat{W}}_{j,k}^{}+\Delta {\hat{W}}_{i,k}^{ο}\cdot {\hat{W}}_{j,k}^{\mathrm{true}}\approx {\hat{W}}_{i,k}^{ο}\cdot \Delta {\hat{W}}_{j,k}^{}+\Delta {\hat{W}}_{i,k}^{ο}\cdot {\hat{W}}_{j,k}^{}$$

For detailed derivation of measurement noise and covariance in this section, they are given explicitly in Appendix A.

### 2.3. Calibration Method of θ

Assuming that the star camera has $n_{s,k}$ observed vectors at the time k, and the remote sensing camera has $n_{c,k}$ observed vectors, so the number of $z_{ij,k}$ is $n_{s,k}n_{c,k}$.

To build a measurement vector Ζ that has $n_{s,k}n_{c,k}$ elements.

(23) $$Z\equiv {\left[z_{11,k},...,z_{n_{i}n_{j},k}\right]}^{T}$$

So, we get the measurement equation: $Z_{k}=H_{k}\theta +\Delta Z_{k}$.

$H_{k}$ can be built from the Equation (21) and $\Delta Z_{k}$ is gaussian white noise sequence with the covariance of $P_{Z_{k}}$.

Using the theory of maximum likelihood estimation [19,20], the negative log-likelihood function of the maximum likelihood estimation θ is:

(24) $$J_{\psi }\left(\theta \right)=\frac{1}{2}\sum_{k=1}^{N}\left[{\left(Z_{k}-H_{k}\theta \right)}^{T}P_{Z_{k}}^{-1}\left(Z_{k}-H_{k}\theta \right)+\log\det P_{Z_{k}}+\log2\pi n_{sk}n_{ck}\right]$$

We solve the minimum value of the above equation and get the normal equation.

(25) $$\begin{array}{l} P_{\theta \theta }^{-1}\theta^{*}=\sum_{k=1}^{N}H_{k}^{T}P_{Z_{k}}^{-1}Z_{k}\\ P_{\theta \theta }^{-1}=\sum_{k=1}^{N}H_{k}^{T}P_{Z_{k}}^{-1}H_{k} \end{array}$$

$P_{\theta \theta }^{}$ is the covariance matrix of the estimation error. The relative conversion error can be estimated from the above equation.

For detailed of redundancy problem of θ calculation, decomposition algorithm and star vector measurement error, we have written explicitly in Appendix B.

To explain the method more clearly, the following will explain the specific steps:

① Obtain the measurement vector ${\hat{U}}_{i,k}^{}$ and uncalibrated installation matrix $R_{CS}^{0}$, then we get the ${\hat{W}}_{i,k}^{ο}$ in uncalibrated remote sensing camera coordinate system;
② Calculate angle cosine error $z_{ij,k}$;
③ For each k moment, construct the $n_{s,k}n_{c,k}$ measurement vector $Z_{k}$;
④ Calculate the measurement sensitivity matrix $H_{k}$, and its dimension is $n_{s,k}n_{c,k}\times 3$;
⑤ Calculate $R_{{\hat{W}}_{i,k}^{}}$ and $G_{{\hat{W}}_{i,k}^{}}$;
⑥ Calculate $B_{k}$ and then get $U_{k}$, $S_{k}$ by singular value decomposition (SVD). Determine the number of singular values $l_{\max}$;
⑦ Calculate $\zeta_{k}$ and $C_{k}$;
⑧ Preserve the first $l_{\max}$ line to get ${\tilde{\zeta }}_{k}$, ${\tilde{C}}_{k}$ and ${\tilde{S}}_{k}$;
⑨ Take the above variables into the measurement equation to get θ and $P_{\theta \theta }$;
⑩ Calculate the error matrix M and installation matrix.

Repeat steps ① to ⑩ until the error matrix approaches the unit matrix. Generally, only one iteration can achieve the effect. The definition of $B_{k}$, $U_{k}$, $S_{k}$, $\zeta_{k}$, $C_{k}$ and etc. can be found in Appendix B.

In this paper, a uniform estimate method of the installation matrix is proposed. It can be used to estimate error matrix M of spacecraft sensors on orbit. Firstly, a statistical model of the measurement error of the star camera is proposed. A set of attitude independent measurements can be obtained; here means angle cosine of observation vectors are obtained. Based on these measurements, the error estimation is obtained. To facilitate numerical calculation, a decomposition algorithm is proposed.

It should be noted that the calculation of $z_{ij,k}$ contains two similar variable subtractions, the result’s significant digits are less than the original vectors’. When the result of variables subtraction is the order of arc-second and nearly 6 significant figures are lost. Therefore, double precision should be used in calculations. Besides, the first-order terms are retained only in the linearization process. Besides, the proportional non-linear error of ${\left|\theta \right|}^{2}$ can be eliminated by iterative methods. Finally, because the mean of $z_{ij,k}$ is zero, it is good for removing gross error through calculating variance.

## 3. Algorithm Simulation

### 3.1. Virtual CCD Sensor Model

**Figure 2:**
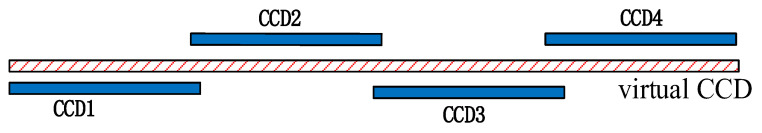
The diagram of Virtual CCD and real CCD relative position.

In the actual data processing, we obtain the images from four independent CCDs which have different rigorous sensor models. Too many models bring too much calculation consumption. Therefore, the four original CCD images are spliced into a virtual image, it can avoid the effect of internal and external errors from the original image (see Figure 2). The virtual imaging CCD has the following advantages [21]:

① There is no lens distortion, so it is an ideal pinhole imaging method to eliminate the lens distortion caused by multi CCDs;
② The virtual CCD is a single line array located on the focal plane which can eliminate the nonlinear distortion of multi CCDs and the splicing distortion of multi CCDs, and tilt distortion of CCDs also can be eliminated;
③ In reality, the pixel size of real CCDs is different, and the virtual CCD pixel size is uniform and easy to calculate.

To better reduce the location error of virtual CCD, we use the internal orientation elements to calculate direction angle $\left(\varphi_{xi},\varphi_{yi}\right)$ to the pixel $\left(x_{i},y_{i}\right)$ on the ideal imaging surface. Repeat the above step to bring all the actual pixels into the calculation, then use the least square method to find the best fitting line as the position of virtual CCD. The expression is y=ax+b: where a and b are unknown numbers, the observation equation is established:

(26) $$V_{}=Ax-L_{}$$

where $A={\left[\begin{array}{cc} x_{1} & 1\\ \vdots & \vdots \\ x_{n} & 1 \end{array}\right]}_{n\times 2},L={\left[\begin{array}{c} y_{1}\\ \vdots \\ y_{n} \end{array}\right]}_{n\times 1},x=\left[\begin{array}{c} a\\ b \end{array}\right]$, n is pixel number.

So, we define the ideal position is:

(27) $$x={\left(A^{T}A\right)}^{-1}A^{T}L$$

$x\in \left[x_{1},x_{n}\right]$, $x_{i}$ is the uniform distribution pixel position of the virtual CCD.

Because of the change of the real light direction, and the based high surface is changed, so it will import elevation error. In this paper, four TDI (Time Delayed and Integration) CCDs are proposed to be installed by a splicing reflector. Four CCDs are installed by overlapping. The splicing accuracy is better than 2μm. The focal length of the remote sensing camera is set at 10m and the resolution along the orbit is 0.5 m. The 1250 km elevation error may cause a 0.5-pixel offset on the image plane [22]. This shows that the virtual imaging based on the average elevation reference plane can realize the seamless splicing of four CCDs, which provides the theoretical basis for the following calculation.

### 3.2. Simulation Model

Firstly, the orbit model is established to generate six elements of orbit data $\omega_{i}$ (the external orientation elements), and the data frequency is 1Hz [23].

Select N calibration points from the whole virtual image and records the image coordinates. According to the virtual CCD sensor model and Digital Elevation Mode (DEM) data, we obtain the corresponding three-dimensional space coordinates of the ground points by iteration. At the same time, we record the select the calibration point’s row number and calculate the exposure time $t_{i}$ of each calibration point. For linear pushbroom CCD, the exterior orientation elements and satellite camera attitude of each scan row are continuously changing. Therefore, if the satellite moves slowly and smoothly, the exterior orientation line elements can be obtained by polynomial interpolation or general polynomial fitting. Generally speaking, there is no significant difference between the second-order general polynomial and the eighth order Lagrange polynomial interpolation results, but on the premise of obtaining high-precision exterior orientation elements, the linear part and high-frequency part of system error can be separated by the second-order polynomial which can better reflect the actual physical significance [24].

**Figure 3:**
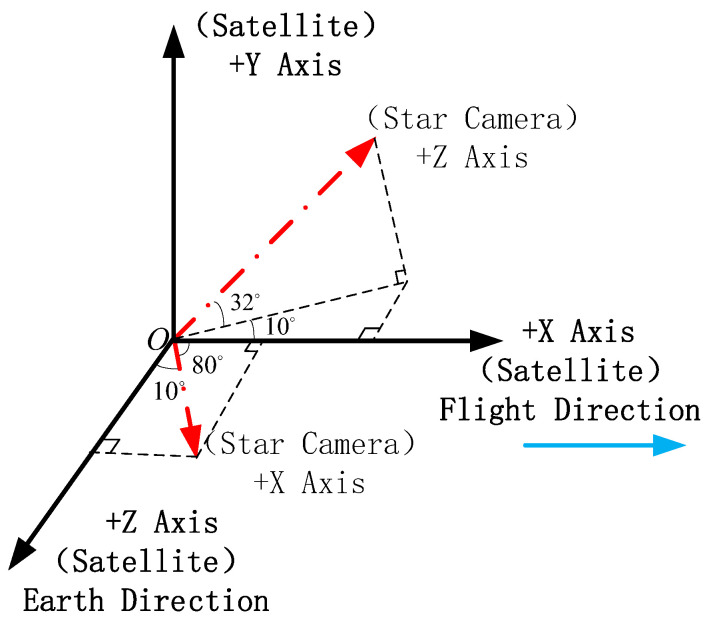
The installation relationship of satellite camera and satellite.

Set the installation relationship of the star camera and remote sensing camera as shown below:

To facilitate discussion, we define the satellite’s *Z*-axis as the optical axis of the remote sensing camera. The theoretical quaternion of the star camera is calculated according to the orbit data of the above, provided in the previous paragraph, and the theoretical installation relationship provided in Figure 3. These quaternion data are used to calculate the star image from the virtual star camera. Star image is represented by 0~255 gray level, and gaussian white noise with zero mean is added as image noise.

### 3.3. Simulation Verification

The simulations proposed in this paper are under different levels of noise. These calculations were implemented in MATLAB in the Microsoft Windows environment on Quad-Core i7 4.0 GHz PC. The geo-position errors decomposition is shown in Table 1. The star camera and the remote sensing camera parameters are shown in Table 2 and Table 3.

The distortion model of the star camera refers to the Equations (4)–(6). The radial distortion (q_1_, q_2_) and tangential distortion (p_1_, p_2_) are all displayed in the first and second order. The distortion values refer to the laboratory calibration results of the star camera. The remote sensing camera starts to number the pixels from the starting point of the CCD. We define the principal point of the CCD as a no distortion pixel. As the distance between each pixel and the principal point increases, the distortion also increases. The sampling pixels and corresponding distortion values are shown in Table 3. The orbit of the remote sensing satellite is the sun-synchronous orbit, the orbit altitude is 530 km. The GPS positioning accuracy is 2 m. The vibration error of the satellite platform is 0.1″. The asynchronous error of satellite clock is 0.1 ms. The distortion calibration residual of remote sensing camera is set to 0.3 pixels [25]. In order to more realistically simulate the motion of the remote sensing camera on orbit, we use the real motion data of the remote sensing camera on-orbit as the motion simulation. 

The Table 1 shows the partial simulation geo-positioning errors. Table 2 and Table 3 show the parameters of star camera and remote sensing camera. The distortion term of the remote sensing camera refers to the distortion of the sampling pixels in each distance with the CCD starting point as the origin.

**Figure 4:**
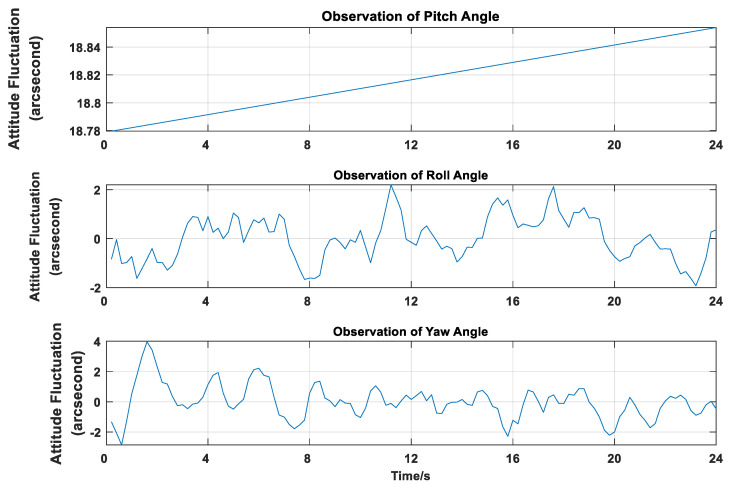
Imaging Attitude Observations of the Remote Sensing Camera.

It is worth mentioning that in actual situations, the pitch and roll angles of the remote sensing camera show an oscillating waveform rather than static: please see Figure 4. In order to show the attitude changes better, the position 0 in Y-ordinate is the angle mean. We capture the observation data for 24 s as a display.

The triaxial deviation of the star camera and remote sensing camera installation matrix is set to [−0.003, 0.002, 0.001], and the unit is the radian. Therefore, the deviation matrix is:

(28) $$M=\left[\begin{array}{ccc} 1 & 0.001 & -0.002\\ -0.001 & 1 & 0.003\\ 0.002 & -0.003 & 1 \end{array}\right]$$

We calculate the star information in the virtual field of view based on the attitude of the star camera. The attitude is obtained from the simulation. We use star information to generate virtual star images. By adding gaussian image noise to the background of the virtual star image, the accuracy of the centroiding can be controlled. 

For the star camera’s centroiding error, the general error is about 0.05 pixel. The accuracy of the star camera’s centroiding is $\sigma_{s}$ pixel and $\sigma_{s}\in \left[0.05,0.10\right]$. The selected GCT error in the remote sensing camera’s image obeys the gaussian zero mean and the standard deviation of $\sigma_{c}$ pixel, $\sigma_{c}\in \left[0.1,1.0\right]$ [26]. Here we define a complete exposure period of the remote sensing camera as one frame.

#### 3.3.1. Performance and Robustness of the Algorithm

In order to show the calculation error of installation matrix clearly, we use the three-axis deviation angle as the result of Euler angle transformation of deviation matrix through Z-X-Y rotation orders in the following discussion.

**Figure 5:**
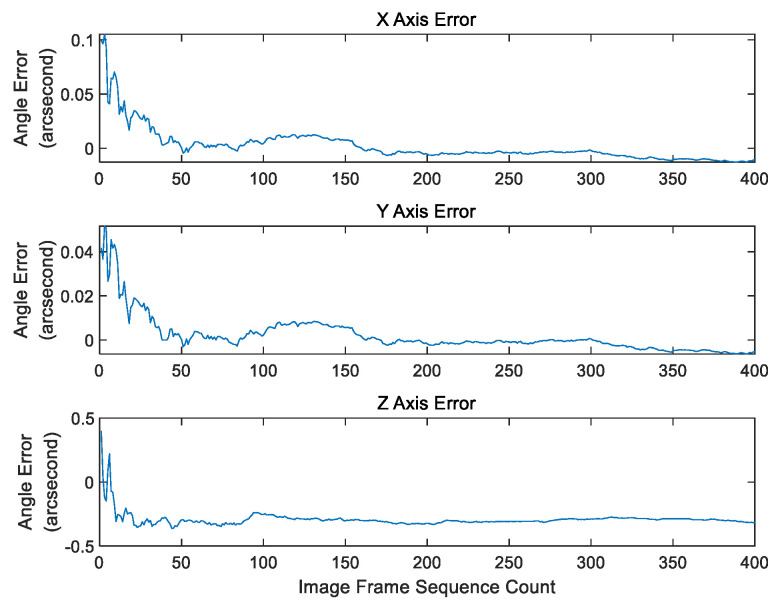
Deviation angle error(arc-second) estimation with frame in $\sigma_{s}=0.06,\sigma_{c}=0.1$ (pixel).

**Figure 6:**
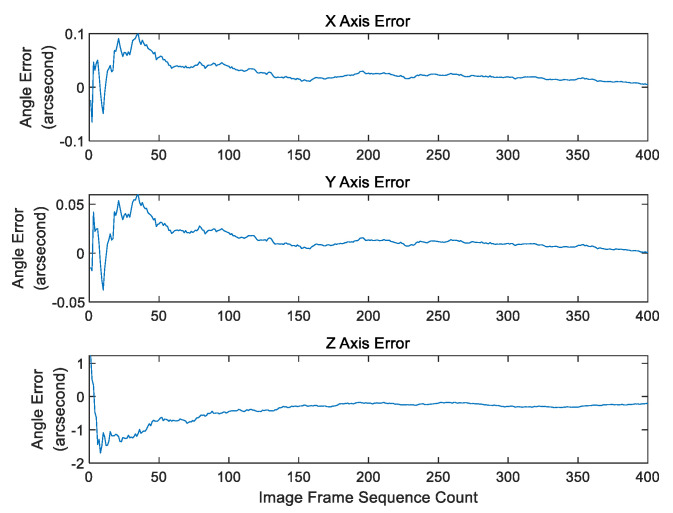
Deviation angle error (arc-second) estimation with frame in $\sigma_{s}=0.10,\sigma_{c}=1.0$ (pixel).

For example, when $\sigma_{s}=0.06,\sigma_{c}=0.1$ (pixel), the figure below is the error between the calculated three-axis deviation angle and the truth deviation angle, the unit is arc-second. It can be clearly seen that when after about 150 frames, the X and Y axis error tends to 0, and the error estimation of the rolling axis is about 0.2 arc-second. Even in the case of large error, such as $\sigma_{s}=0.10,\sigma_{c}=1.0$ (pixel), the estimation of deviation angle converges very fast, and the estimation error of the rolling axis is about 0.5 arc-second. For the efficiency of the algorithm in 400 frames of data, if S_k_ (Appendix B) is less than the set threshold, it will be judged that it has converged. Obviously, it is not the final convergence result of the algorithm. Because the error of *z*-axis is 5–10 times larger than that of *x*-axis and *y*-axis, when the error of *x*-axis and *y*-axis have satisfied the convergence condition, the error of z axis has not approached to 0. Therefore, it can be seen from Figure 5 and Figure 6 that this method can converge to the ideal result after 150 frames.

**Figure 7:**
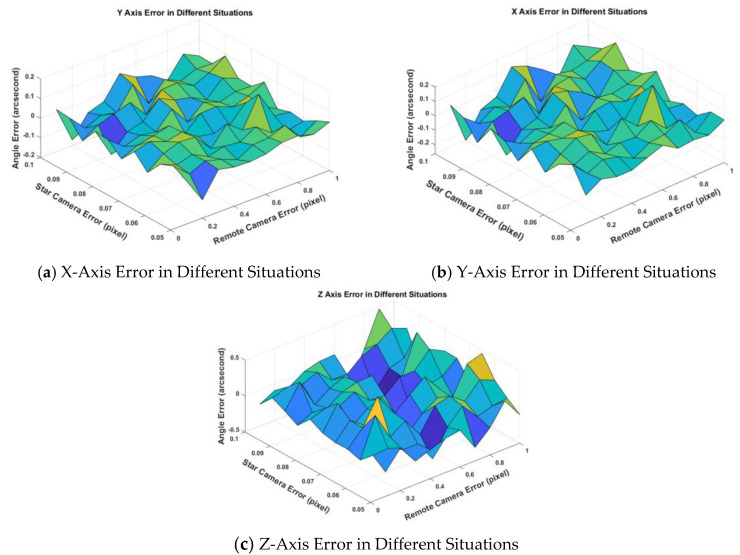
Triaxial deviation angle of calculated installation matrix under different errors of the star camera and remote sensing camera by using this paper algorithm. The unit of centroiding error is pixel for X and *Y*-axis and arc-second for *Z*-axis.

Test the robustness of this method under a different centroiding error of the star camera and remote sensing camera. The range of centroiding error which we tested is $\sigma_{s}\in \left[0.05,0.10\right],\sigma_{c}\in \left[0.1,1.0\right]$ (pixel). In each case, the error of triaxial after convergence is shown as follows in Figure 7:

It can be seen from the above three figures in Figure 7 that the accuracy of the method for calculating the installation matrix is accurate and stable. When setting different centroiding errors of the star camera and remote sensing camera, the triaxial estimation error’s mean value is [−0.0018,−0.0001,−0.2602], for X, Y and Z axis in arc-second. The estimation error’s standard deviation is [0.0159,0.0105,0.0927] in arc-second.

#### 3.3.2. Comparison with the Wang’s Algorithm

Wang’s calibration algorithm for the on-orbit installation matrix calibration is introduced here for comparison [13]. The difference between the two algorithms is that Wang’s algorithm needs the attitude from the star sensor, and he calculates the transformation of the star sensor, remote sensing camera, satellite and satellite orbit coordinate systems. It obtains the motion matrix $R_{co}$ which represents each remote sensing image relative to the orbit coordinate systems through a data iteration. Finally, the installation matrix is solved easily. Different from the method in this paper, Wang’s method can only calculate the installation matrix independently for each frame of image, but it cannot directly increase the number of frames to improve the accuracy of the method.

**Figure 8:**
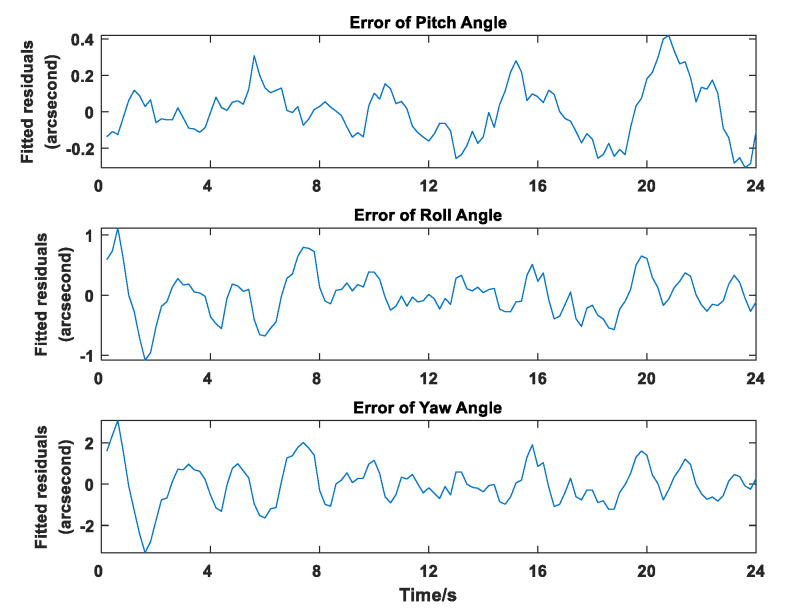
Residual Results of Attitude Fitting.

Wang uses a third-order polynomial to fit the motion state of the remote sensing camera. The residuals of three angles after fitting are as shown in Figure 8. We can see that the high-order polynomials cannot accurately describe the motion of the remote sensing camera, even if we increase the order of the polynomials from the 5th to the 7th, there is no significant improvement. That shows that in practical situations it is inaccurate to use a polynomial method to describe the time-varying state of a remote sensing camera. In addition, Wang’s method only solves polynomials for each remote sensing image. As the order of polynomials increases, the coefficients to be solved also increase, which requires more calculation points.

Then, we show the schematic diagram of the deviation angle error calculated by Wang’s algorithm under different error conditions.

**Figure 9:**
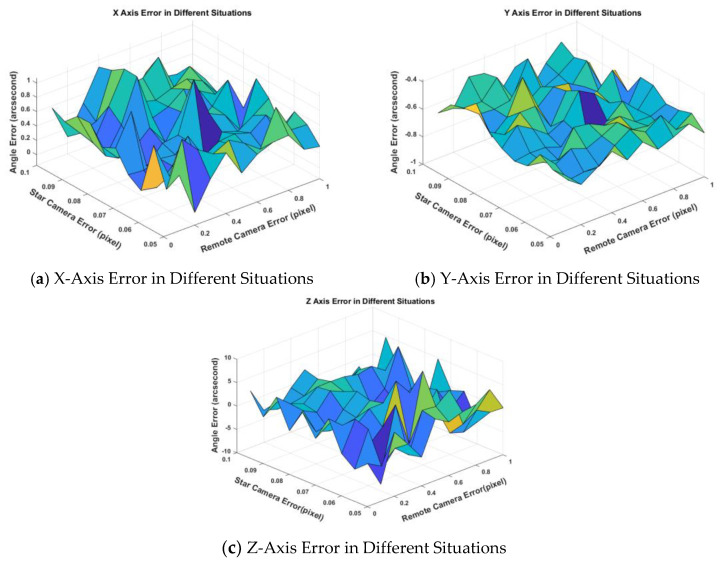
Triaxial deviation angle of calculated installation matrix under different errors of star camera and remote sensing camera by using the Wang’s algorithm. The unit of centroiding error is pixel for X and *Y*-axis and arc-second for *Z*-axis.

We can compare Figure 9 with Figure 7 and find that the deviation angle error of Wang’s algorithm is larger than the result of the method in this paper.

**Figure 10:**
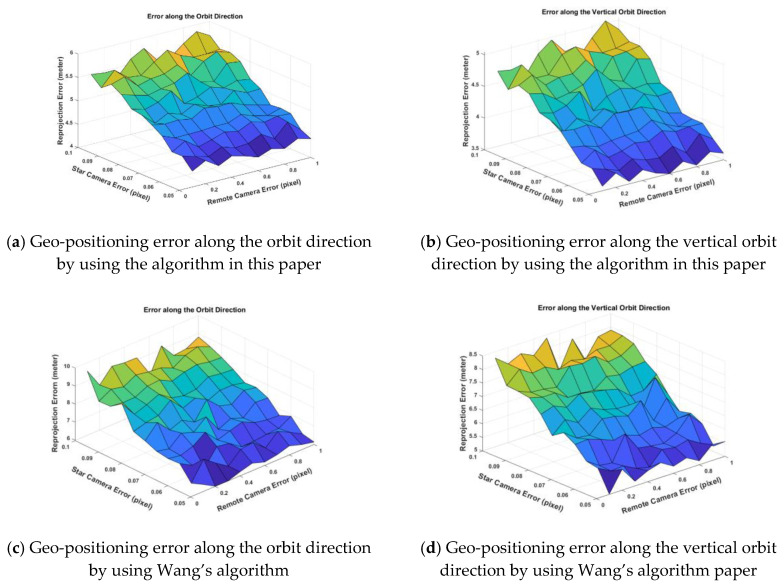
The mean value of the geo-positioning error of test ground points after on-orbit correction of installation matrix. The unit of geo-positioning error is the meter for *Z*-axis.

In Table 4, we can see in the case of $\sigma_{s}=0.07,\sigma_{c}=0.5$ (pixel), the angle error of three-axis is reduced by 5–14 times. However, in the case of $\sigma_{s}=0.10,\sigma_{c}=1.0$ (pixel), the angle error of three-axis is reduced more than 10 times. We can also see that the error of the deviation angle does not change significantly with the increase of the noise of the star camera and remote sensing camera by using the paper algorithm. The reason is we use a joint calculation method with multi-frame data, and the random error of the image points in the star camera and the remote sensing camera will not affect the calibration of the installation matrix. Since the input error is added in the simulation process, the error of *x*-axis and *y*-axis fluctuates around 0. The error of the Z axis is about 5–10 times that of the x -axis and *y*-axis, which is related to the low accuracy of the star camera Z axis. The calculation error of the installation matrix directly leads to the decline of the final geo-positioning accuracy. The schematic diagrams using the same verification point to the geo-position of two algorithms and are shown in Figure 10.

Figure 10 shows that with the decrease of the location error of the star camera’s centroiding, the positioning accuracy of the geo-positioning also decreases. When the centroiding accuracy of star camera is 0.05 pixel, the attitude accuracy of the star camera is about 0.5″ (3σ). 

Let us combine Table 5 with Figure 10. When we set the $\sigma_{s}=0.05,\sigma_{c}=0.3$ pixel, there is a total of seven real remote sensing images (6144 pixels × 26000 pixels × 4 pieces), and the whole star images are generated by the calculation of the virtual field of view. In total, 20 ground test points are selected from one image, and the experiment of geo-positioning accuracy is carried out with the algorithm. It can be seen that the geo-positioning RMSEs (root mean square errors) in planimetry along the orbit direction and the vertical orbit are 4.32 m and 3.54 m which is better than the 5.96 m and 5.17 m using Wang’s method. Combine the results between the two methods, and this paper method is about 2.5 m less than Wang’s method on average, which is related to the accuracy improvement of the calculation of the installation matrix by using the paper method.

Furthermore, the reason for the large geo-positioning error of Wang’s is each set that composed of a star image and remote sensing image is calculated independently, and one set only provides one installation matrix $R_{sc}$. This calculation method leads to high coupling between $R_{co}$ and $R_{sc}$ (Refer to Equation (17)). The $R_{co}$’s accuracy directly leads to the final $R_{sc}$’s accuracy. We can see in Figure 8, the $R_{co}$’s model cannot fit well which causes the residual error. This error couples to $R_{sc}$ directly.

## 4. Conclusions

In this paper, a novel algorithm on-orbit for installation matrix estimation is proposed. This method can realize the on-orbit calibration of the installation matrix accurately. We can use the ultra-high precision star camera as the measuring instrument for the external orientation elements measurement and then realize the high-precision geo-positioning. This method has the following characteristics: (1) To realize the high precision earth observation by using a star camera, we should calculate the installation matrix between the star camera and the remote sensing camera firstly. When the remote sensing camera’s external orientation elements are determined, the vector angle between the GCT vector and the star vector is deduced as a rotation invariance. (2) For the data redundant of measurement vectors, we propose a batch processing algorithm based on SVD, which is used to process long-term data. This method is not sensitive to short-term data loss and is suitable on orbit. This can make full use of multi-frame data to improve the accuracy of the installation matrix calculation

This method can use most information of the star vector and the GCT vector, and it avoids the complicated satellite on-orbit movement compensation which impedes the estimation of the installation matrix. At the same time, a batch processing algorithm based on SVD is added to enhance the on-orbit data processing ability and robustness of the algorithm. The simulation results demonstrate that the method can effectively improve the accuracy estimation of the installation matrix. In a wide range of sensor noise, the error of the X, *Y*-axis is about the order of 10^−2^ arc-second. Then, we achieve a high-precision geo-positioning measurement by using the above result. By comparing it with Wang’ on-orbit algorithm, we have improved the ground geo-positioning accuracy by above 20%. So, we have achieved an algorithm that only needs simple data on-orbit, with high calculation accuracy and strong robustness. It is also suitable for an on-orbit multi-frame joint calculation. It provides better theoretical support for future photogrammetry without GCPs. Future work will obtain more complete practical results of the on-orbit data to verify the actual effect of the algorithm and apply the algorithm to remote sensing satellites to evaluate the long-term performance under on-orbit conditions.

## Figures and Tables

**Table 1:** Geo-Positioning Errors Decomposition.

Error Items		Geo-Positioning Errors/ meter
Name	Value	
GPS positioning accuracy	2 m	2
vibration of the satellite platform	0.1″	0.25
asynchronous error of satellite clock	0.1 ms	0.75 (the orbit direction)
distortion calibration residual of remote sensing camera	0.3 pixel	0.21

**Table 2:** Star Camera Parameters.

Focal Length (mm)		100
Field of View (°)		$18^{\circ }\times 14^{\circ }$
Data Rate (Hz)		5
Principal Point (Pixel)		(2560,1920)
Pixel Size (μm)		6.4
Distortion	p1	2.29 ∗ 10^−5^
	p2	−2.96 ∗ 10^−5^
	q1	−9.30 ∗ 10^−7^
	q2	−4.25 ∗ 10^−9^

**Table 3:** Remote Sensing Camera Parameters.

Focal Length (m)	10	
CCD Pixel	6144 each	
CCD Pixel Size (m)	$1\times 10^{-5}$	
Principal Point (Pixel)	3067	
Distortion (Pixel, μm)	56	1077.2
	203	866.3
	…	…
	3067	0
	5784	892.1
	5912	1082.7

**Table 4:** The Deviation Angle Error of Different Parameter.

$\mathrm{Camera}\;\mathrm{Error}\;[\sigma_{s},\sigma_{c}]$ (Pixel)	Wang’s Algorithm the Deviation Angle Error			Paper Algorithm the Deviation Angle Error		
	X (″)	Y (″)	Z (″)	X (″)	Y (″)	Z (″)
0.06,0.3	0.3198	−0.5958	−7.4917	0.0383	0.0202	−0.2788
0.07,0.5	0.3644	−0.6686	4.4221	−0.0640	−0.0464	−0.5072
0.08,0.5	0.1656	−0.7332	4.7857	−0.0809	−0.0441	−0.1987
0.09,0.7	0.5519	−0.6898	−8.4521	−0.0064	0.0038	−0.6839
0.10,1.0	0.1837	−0.7338	−9.6278	0.0121	0.0135	0.6246

**Table 5:** Geo-Positioning Results of the Different Algorithm.

Camera Error /Pixel		Orbit Direction/Meter		Vertical Orbit Direction/Meter	
$\sigma_{c}$	$\sigma_{s}$	**Wang**	**The Paper**	**Wang**	**The Paper**
0.30	0.05	5.96	4.32	5.17	3.54
0.30	0.055	6.65	4.41	5.56	3.64
0.30	0.060	6.31	4.53	5.82	3.77
0.30	0.065	6.97	4.71	6.20	3.87
0.30	0.070	7.39	4.92	6.24	4.15
0.30	0.075	7.25	4.98	6.46	4.18
0.30	0.080	7.73	5.12	6.84	4.31
0.30	0.085	8.02	5.18	7.18	4.34
0.30	0.090	8.13	5.29	7.16	4.49
0.30	0.095	8.39	5.38	8.57	4.53
0.30	0.010	9.23	5.29	8.11	4.48

## Appendix A

Suppose $\Delta {\hat{U}}_{i,k}$ is the gaussian white noise with zero mean and covariance of $R_{{\hat{U}}_{i,k}}$, that means $\Delta {\hat{U}}_{i,k}\sim N(0,R_{{\hat{U}}_{i,k}})$, and it is worth noting that different observation vectors are independent of each other.

(A1) $$E\left\{\Delta {\hat{U}}_{i,k}\Delta {\hat{U}}_{i',k^{\prime }}^{T}\right\}=\delta_{ii'}\delta_{kk'}R_{{\hat{U}}_{i,k}}$$

E {·} is the expectation operator. If the observation vector is a unit vector and the first-order component of $\Delta {\hat{U}}_{i,k}$ is perpendicular to ${\hat{U}}_{i,k}$, then [27],

(A2) $$\Delta {\hat{U}}_{i,k}\cdot {\hat{U}}_{i,k}=0$$

$R_{{\hat{U}}_{i,k}}$ is the singular matrix, so here is:

(A3) $$R_{{\hat{U}}_{i,k}}{\hat{U}}_{i,k}^{\mathrm{true}}=0$$

The above three equations are first-order approximations. Generally, the covariance R is small. This approximation will not affect the estimation of the installation matrix.

Similar to (8), ${\hat{W}}_{i,k}^{\mathrm{true}}$ is the true value of the observation vector, $\Delta {\hat{W}}_{i,k}$ is the measurement noise, Supposing $\Delta {\hat{W}}_{i,k}$ is gaussian white noise with zero mean and covariance of $R_{{\hat{W}}_{i,k}}$, that means $\Delta {\hat{W}}_{i,k}\sim N(0,R_{{\hat{W}}_{i,k}})$.

(A4) $$\begin{array}{c} E\left\{\Delta {\hat{W}}_{i,k}\Delta {\hat{W}}_{i',k^{\prime }}^{T}\right\}=\delta_{ii'}\delta_{kk'}R_{{\hat{W}}_{i,k}}\\ \Delta {\hat{W}}_{i,k}\cdot {\hat{W}}_{i,k}=0\\ R_{{\hat{W}}_{i,k}}{\hat{W}}_{i,k}^{\mathrm{true}}=0 \end{array}$$

We can get $R_{{\hat{U}}_{i,k}}=R_{CS}^{T}R_{{\hat{W}}_{i,k}}R_{CS}$.

For the Equation (19) there is $\Delta {\hat{W}}^{ο}{}_{i,k}\sim N(0,R_{{\hat{W}}^{ο}{}_{i,k}}),R_{{\hat{W}}^{ο}{}_{i,k}}=R_{CS}^{ο}R_{{\hat{U}}_{i,k}}R_{CS}^{οT}$.

For the Equation (20), the derivation process is provided here.

(A5) $$\begin{array}{cc} {\hat{W}}_{i,k}^{ο}\cdot {\hat{W}}_{j,k}^{} & ={\hat{W}}_{i,k}^{\mathrm{true}}\cdot {\hat{W}}_{j,k}^{true}+\left(-{\hat{W}}_{i,k}^{\mathrm{true}}\times {\hat{W}}_{j,k}^{true}\right)\cdot \theta +{\hat{W}}_{i,k}^{\mathrm{true}}\cdot \Delta {\hat{W}}_{j,k}^{}+\Delta {\hat{W}}_{i,k}^{ο}\cdot {\hat{W}}_{j,k}^{\mathrm{true}}+\Delta {\hat{W}}_{i,k}^{ο}\cdot \Delta {\hat{W}}_{j,k}^{}\\ & ={\hat{V}}_{i}^{true}\cdot {\hat{V}}_{j}^{true}+\left(-{\hat{W}}_{i,k}^{\mathrm{true}}\times {\hat{W}}_{j,k}^{true}\right)\cdot \theta +{\hat{W}}_{i,k}^{\mathrm{true}}\cdot \Delta {\hat{W}}_{j,k}^{}+\Delta {\hat{W}}_{i,k}^{ο}\cdot {\hat{W}}_{j,k}^{\mathrm{true}}+\Delta {\hat{W}}_{i,k}^{ο}\cdot \Delta {\hat{W}}_{j,k}^{} \end{array}$$

For $z_{ij,k}$ and $\Delta z_{ij,k}$, it can be seen that the first-order expression of the angle cosine difference has nothing to do with attitude, and the above variables need to satisfy the following constraints.

(A6) $$\begin{array}{l} E\left\{\Delta z_{ij,k}\right\}=0;\\ E\left\{\Delta z_{ij,k}^{2}\right\}=E_{k}(i|j|i)+E_{k}(j|i|j);\\ E\left\{\Delta z_{ij,k}\Delta z_{iu,k}\right\}=E_{k}(j|i|u);\\ E\left\{z_{ij,k}z_{uv,k}\right\}=0;i,j,u,v\;\mathrm{are}\;\mathrm{different} \end{array}$$

There is $E_{k}(i|j|u)\equiv {\hat{W}}_{i,k}^{οT}R_{{\hat{W}}_{j,k}^{ο}}{\hat{W}}_{u,k}^{ο}$.

## Appendix B

### Appendix B.1. Redundancy Problem

Measurement vectors $z_{ij,k}$ are not all independent. Suppose the star camera has $n_{s,k}$ directly observed vectors, remote sensing camera has $n_{c,k}$ directly observed vectors at the k time, so there are $2n_{s,k}+2n_{c,k}$ degrees of freedom. However, there are $n_{s,k}n_{c,k}$ combinations. Since the attitude has three degrees of freedom and $z_{ij,k}$ are independent of the attitude, so there are only $2n_{s,k}+2n_{c,k}-3$ independent $z_{ij,k}$ vectors. When $n_{s,k}n_{c,,k}>2n_{s,k}+2n_{c,k}-3$, $z_{ij,k}$ are redundant and $P_{\theta \theta }$ is a singular matrix. At this time, the Equation (A6) cannot be directly used to estimate the covariance matrix error, so singular value decomposition is needed to calculate the θ.

### Appendix B.2. Decomposition Algorithm

The observation vector error covariance matrix in the remote sensing camera coordinate system can be expressed as:

(A7) $$R_{{\hat{W}}^{}{}_{i,k}}=G_{{\hat{W}}^{}{}_{i,k}}G_{{\hat{W}}^{}{}_{i,k}}^{T}$$

The covariance matrix $R_{{\hat{W}}^{}{}_{i,k}}$ can be decomposed into one matrix left multiply its transport matrix. Here we define this matrix named $G_{{\hat{W}}^{}{}_{i,k}}$. So, the observation vector error can be expressed as:

(A8) $$\Delta {\hat{W}}^{}{}_{i,k}=G_{{\hat{W}}^{}{}_{i,k}}\varepsilon_{i,k}$$

for $E\left\{\varepsilon_{i,k}\right\}=0$ and $E\left\{\varepsilon_{i,k}\varepsilon_{i^{\prime },k}^{T}\right\}=\delta_{ii^{\prime }}I_{3\times 3}$, so we get the equation:

(A9) $$z_{ij,k}=\left(-{\hat{W}}_{i,k}^{ο}\times {\hat{W}}_{j,k}^{}\right)\cdot \theta +B_{ij,k}^{i}\varepsilon_{i,k}^{o}+B_{ij,k}^{j}\varepsilon_{j,k}$$

where $B_{ij,k}^{i}={\left({\hat{W}}_{j,k}^{}\right)}^{T}G_{{\hat{W}}^{ο}{}_{i,k}},B_{ij,k}^{j}={\left({\hat{W}}_{i,k}^{}\right)}^{T}G_{{\hat{W}}^{}{}_{j,k}}$.

In summary, we rewrite the measurement equation as

(A10) $$Z_{k}=H_{k}\theta +B_{k}\varepsilon_{k}$$

$\varepsilon_{k}\equiv {\left[\varepsilon_{1,k}^{oT},...,\varepsilon_{n_{s,k},k}^{oT},\varepsilon_{1,k}^{T},...,\varepsilon_{n_{c,k},k}^{T}\right]}^{T}\sim N\left(0,I_{3(n_{s,k}+n_{c,k})\times 3(n_{s,k}+n_{c,k})}\right)$, $B_{k}$ is a $n_{s,k}n_{c,k}\times 3(n_{s,k}+n_{c,k})$ matrix, $H_{k}$ is a $n_{s,k}n_{c,k}\times 3$ matrix and $P_{Z_{k}}=B_{k}B_{k}^{T}$.

We calculate singular value decomposition (SVD) like $B_{k}=U_{k}S_{k}V_{k}^{T}$. $U_{k}$ and $V_{k}^{}$ are orthogonal matrix obviously, $S_{k}$ is a $n_{s,k}n_{c,k}\times 3(n_{s,k}+n_{c,k})$ diagonal matrix and there is ${\left(S_{k}\right)}_{11}\geq {\left(S_{k}\right)}_{22}\geq ...\geq 0$. So $P_{Z_{k}}=U_{k}S_{k}S_{k}^{T}U_{k}^{T}=U_{k}D_{k}U_{k}^{T}$ and it is a $n_{s,k}n_{c,k}\times n_{s,k}n_{c,k}$ positive semidefinite matrix.

We multiply the Equation (A10) by $U_{k}^{T}$ from left side, so we get:

(A11) $$\zeta_{k}=C_{k}\theta +S_{k}{\varepsilon^{\prime }}_{k}$$

where ${\varepsilon^{\prime }}_{k}\equiv V_{k}^{T}\varepsilon_{k}\sim N\left(0,I_{3(n_{s,k}+n_{c,k})\times 3(n_{s,k}+n_{c,k})}\right)$, and $\zeta_{k}\equiv U_{k}^{T}Z_{k}$, $C_{k}\equiv U_{k}^{T}H_{k}$.

Assuming ${\left(S_{k}\right)}_{11}\geq {\left(S_{k}\right)}_{22}\geq ...\geq {\left(S_{k}\right)}_{l_{\max,k}l_{\max,k}}\geq 0$, so the first $l_{\max,k}$ components of $\zeta_{k}$ are independent of each other. Here, only preserve the first $l_{\max,k}$ lines of $\zeta_{k},C_{k},S_{k}$ matrix and rewrite as ${\tilde{\zeta }}_{k}={\tilde{C}}_{k}\theta +{\tilde{S}}_{k}{\tilde{\varepsilon^{\prime }}}_{k}$. The tilde indicates line truncation.

So, we change the Equation (22) as the following:

(A12) $$J_{\psi }\left(\theta \right)=\frac{1}{2}\sum_{k=1}^{N}\left[{\left({\tilde{\zeta }}_{k}-{\tilde{C}}_{k}\theta \right)}^{T}P_{{\tilde{\zeta }}_{k}}^{-1}\left({\tilde{\zeta }}_{k}-{\tilde{C}}_{k}\theta \right)+\log\det P_{{\tilde{\zeta }}_{k}}+\log2\pi (2n_{c,k}+2n_{s,k}-3)\right]$$

Similar to Equation (26) we get:

(A13) $$\begin{array}{l} P_{\theta \theta }^{-1}\theta^{*}=\sum_{k=1}^{N}{\tilde{C}}_{k}^{T}{\tilde{D}}_{k}^{-1}{\tilde{\zeta }}_{k}\\ P_{\theta \theta }^{-1}=\sum_{k=1}^{N}{\tilde{C}}_{k}^{T}{\tilde{D}}_{k}^{-1}{\tilde{C}}_{k} \end{array}$$

SVD result obtains the largest subset that is sensitive to installation matrix error.

### Appendix B.3. Star Vector Measurement Error

The star vector measurement error can be expressed by the QUEST measurement model, of which the error vector is symmetrical about this vector. The error covariance matrix can be expressed as:

(A14) $$\begin{array}{l} E\left\{\Delta {\hat{U}}_{i,k}\Delta {\hat{U}}_{i,k}^{T}\right\}=\sigma_{i,k}^{2}(I-{\hat{U}}_{i,k}^{\mathrm{true}}{\hat{U}}_{i,k}^{\mathrm{true}}{}^{T})\\ E\left\{\Delta {\hat{W}}_{i,k}^{ο}\Delta {\hat{W}}_{i,k}^{οT}\right\}=\sigma_{i,k}^{2}(I-{\hat{W}}_{i,k}^{ο}{\hat{W}}_{i,k}^{ο}{}^{T}) \end{array}$$

Combine with the Equations (A4) and (A14), and there is:

(A15) $$E_{k}(i|j|u)=\sigma_{j,k}^{2}\left({\hat{W}}_{i,k}^{ο}\times {\hat{W}}_{j,k}^{}\right)\cdot \left({\hat{W}}_{u,k}^{ο}\times {\hat{W}}_{j,k}^{}\right)$$

For the decomposition of measurement error covariance matrix, we can get $R_{{\hat{W}}_{i,k}^{}}=\left(\sigma_{i,k}〚{\hat{W}}_{i,k}^{}〛\right){\left(\sigma_{i,k}〚{\hat{W}}_{i,k}^{}〛\right)}^{T}$, then $B_{ij,k}^{i}={\left({\hat{W}}_{j,k}^{}\right)}^{T}\left(\sigma_{i,k}〚{\hat{W}}_{i,k}^{ο}〛\right)=\sigma_{i,k}{\left({\hat{W}}_{i,k}^{ο}\times {\hat{W}}_{j,k}^{}\right)}^{T}$, $B_{ij,k}^{j}=\sigma_{j,k}{\left({\hat{W}}_{j,k}^{}\times {\hat{W}}_{i,k}^{ο}\right)}^{T}$.
